# Conservative treatment of 3-part and 4-part proximal humeral fractures: a systematic review

**DOI:** 10.1186/s13018-020-01880-7

**Published:** 2020-08-24

**Authors:** Manuel Soler-Peiro, Lorena García-Martínez, Luis Aguilella, Marcelino Perez-Bermejo

**Affiliations:** 1 Upper Extremity Unit, Orthopaedic Surgery Service, Hospital Universitario de La Ribera, Alzira, Valencia, Spain; 2 School of Medicine, Catholic University of Valencia, Valencia, Spain

**Keywords:** Proximal humeral fracture, Conservative treatment, Outcomes, Constant score, Fracture consolidation, Complications, Neer classification, Review, Malunion, Nonunion, Avascular necrosis

## Abstract

**Background:**

Although there are numerous publications about surgical treatment of proximal humeral fractures (PHFs), few assess conservative treatment, which is the most common approach. The aim of this systematic literature review was to assess criteria for indications, treatment protocols, and outcomes obtained with the conservative treatment of 3-part and 4-part PHF.

**Methods:**

We searched the PubMed and Cochrane databases for clinical studies published between 2000 and 2019 on conservative treatment for 3-part and 4-part PHF that included patients older than 18 years, a minimum follow-up of 1 year, fracture classification, and description of outcomes with assessment scales.

**Results:**

The search yielded 26,660 records. We reviewed 44 of them in full, and finally 6 studies were included. We obtained a population of 133 patients (79% women), with a mean age of 74.3 years (range 25 to 98) and mean follow-up of 32 months (range 12 to 68.8). According to the Neer classification system, there were 41% (55) three-part fractures and 59% (78) four-part fractures; 5.81% of the patients were lost to follow-up. The mean Constant score was 64.5 for three-part fractures and 54.9 patients with four-part fractures. Consolidation was achieved in 95% of the three-part fractures and 91% of the four-part fractures. Loss of mobility varied according to the type of fracture. Regarding complications, the most frequent was malunion (21%), followed by avascular necrosis (9%).

**Conclusions:**

Our data show that most three-part PHFs treated conservatively achieve fracture consolidation even noting a negligible rate of malunion got fair–good functional results with few complications, while the orthopedic four-part PHF treatment presents high rate of consolidation with less rate of malunion than the three-part PHF but achieve poor functional results with few complications.

**Level of evidence:**

Level IV, Systematic Review

## Background

There is a high incidence of proximal humeral fractures (PHFs) in the current population due to the increasing life expectancy and the consequent rise of osteoporotic bone fractures [1]. These fractures are the fourth most common in the elderly population, representing about 6% of all fractures in adults [2]. Care for this pathology consumes a considerable and growing quantity of health system resources.

There are numerous publications on the different surgical treatment options for these fractures, but few studies assess conservative treatment, which is the most frequently used [3], since many of these fractures are minimally displaced and can be treated nonoperatively [4, 5]. Moreover, new methods of osteosynthesis and prosthetic substitution have led to an increase in indications for surgical treatment of displaced PHFs [6], despite the significant rate of complications associated with surgery for these injuries [7]. Recent studies like PROFHER (PROximal Fracture of the Humerus: Evaluation by Randomisation) [8] have stirred controversy around the need to intervene in many of these displaced PHFs.

It should also be mentioned, when treating this type of fracture, the difference between referral hospitals, with well-established shoulder units, and smaller hospitals where the orthopedic surgeons are not subspecialized. Probably, due to the technical complexity of these surgeries, conservative treatment is chosen more frequently in these latter hospitals, while in shoulder units, surgical treatment is more often preferred. For this reason, it is even more necessary to find a consensus when treating PHFs.

This systematic literature review has the following aims: to assess criteria for indications, treatment protocols, and outcomes obtained with conservative treatment of 3-part and 4-part PHFs.

## Methods

### Data sources

We performed a systematic review of publications on conservative treatment for three-part and four-part PHFs, in compliance with the PRISMA protocol [9]. Two review authors independently screened abstracts retrieved from PubMed and the Cochrane Library, and selected articles for full-text assessment. The search was restricted to studies in human participants and published in English from the year 2000 to December 2019. Date last searched was January 20th, 2020. We used the following medical subject headings (MeSH): “Shoulder fracture conservative treatment”, “Shoulder fracture non operative treatment”, “Shoulder fracture non surgical treatment”, “Shoulder fracture outcome”, “Proximal humerus fracture conservative treatment”, “Proximal humerus fracture non operative treatment”, “Proximal humerus fracture non surgical treatment”, “Proximal humerus fracture outcome”, “Proximal humeral fracture conservative treatment”, “Proximal humeral fracture non operative treatment”, “Proximal humeral fracture non surgical treatment”, “Proximal humeral fracture outcome”, “Humerus head fracture conservative treatment”, “Humerus head fracture non operative treatment”, “Humerus head fracture non surgical treatment”, and “Humerus head fracture outcome”.

### Study selection

The inclusion and exclusion criteria were agreed by the authors to obtain a minimal homogeneous information in order to carry out a review.

Inclusion criteria for the articles were (1) studies of three-part and four-part PHFs of traumatic origin (studies excluded pathological fractures) treated conservatively; (2) patients older than 18 years; (3) studies with levels I–IV evidence; (4) studies with minimum follow-up of 1 year; (5) studies with more than 15 participants; (6) studies using a fracture classification system (Neer or AO) (7); studies reporting outcomes on an assessment scale; and (8) studies describing range of motion, pain, and complications (Table 1).

**Table 1:** PICOS criteria for inclusion in the systematic review

Acronym	Definition	Application of the criteria on the present study
P	Participants	Adults with three-part and four-part proximal humeral fractures with traumatic origin
I	Intervention	Conservative treatment
C	Control	None
O	Outcomes	Constant score, complications, range of movement
S	Study design	Studies published between 2000–2019, with levels I–IV, patients older than 18 years, at least 15 patients, minimum follow-up 1 year, studies using a fracture classification system (Neer or AO)

We excluded studies that (1) focused exclusively on surgical treatment for PHF, (2) were systematic reviews and meta-analyses, and (3) reported level V evidence.

### Data extraction

We classified all records retrieved in the search as relevant, potentially relevant, and irrelevant. We extracted from the relevant studies the following data: study design, year of publication, journal of publication, type of study, level of evidence, number of patients, mean age, sex, laterality of fracture, indication for conservative treatment, type of fracture, follow-up, losses, type of conservative treatment, Constant scale, other scales, subjective results, consolidation, and complications. This piloted form was evaluated in duplicate by independent authors.

Studies reporting on patients treated surgically were included only if data on participants treated conservatively were clearly differentiated, and those with inconsistent or ambiguous data were excluded. We resolved any disagreements with two independent reviewers by consensus or adjudication by the senior author.

All institutional and author information were concealed to minimize reviewer bias. The risk of bias was evaluated as suggested by Furlan et al. [10]. The risk of bias was considered to be low when 6 or more criteria out of 12 were met, and the risk was rated as high when less than 6 out of 12 criteria were met.

## Results

**Fig. 1:**
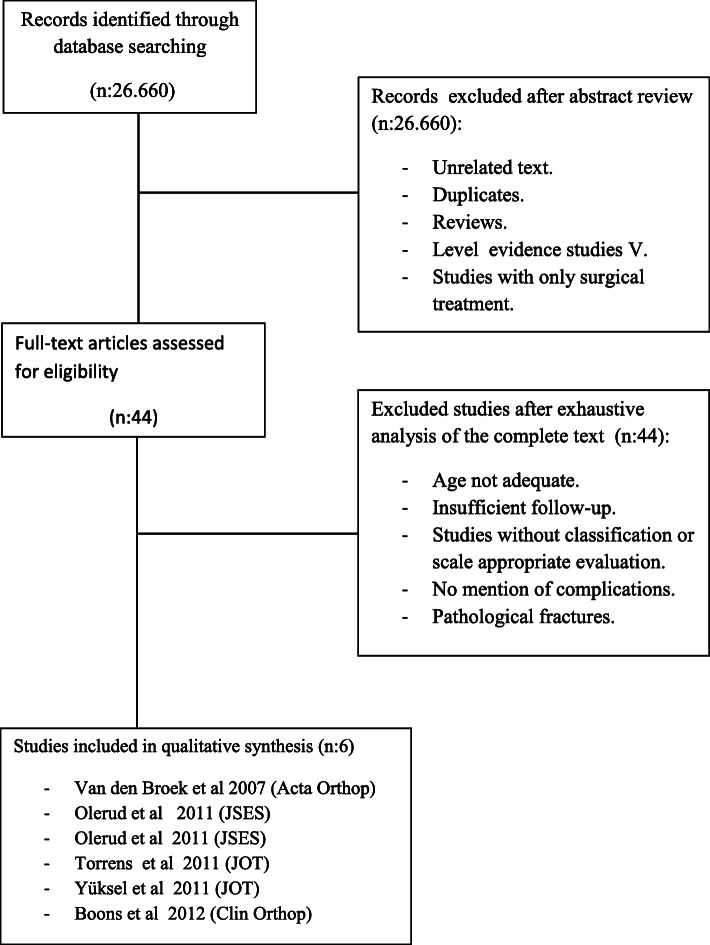
Flow chart of literature search and study selection

Our search strategy yielded 26,660 records. After screening abstracts, the number of potentially relevant records dropped to 44. Full-text assessment further reduced the number of articles that met the inclusion criteria to 6 (Fig. 1). These studies involved a total of 133 patients; three studies produced level I evidence, one study, level II, and two studies, level IV. All included patients who had PHFs with three and four parts, according to the Neer classification. Of the participants, 79% were women, and 21% were men. Mean age at the time of the fracture was 74.3 years (range 25 to 98), and mean follow-up was 32 months (range 12 to 68.8) (Table 2).

**Table 2:** Demographic data

Author	Ev. level	Patients	Average age	Sex	Laterality	Mean follow-up	Losses
Van den Broek et al. 2007 [11] *Arch Orthop Trauma Surg*	II	16	64.4	87.5% F 12.5% M	37.5% R62.5% L	68.8 months	0%
Olerud et al. 2011 *JSES* [12]	I	29	74.9	82.75% F 17.25% M	45% DA	2 years	10,34%
Olerud et al. 2011 *JSES* [13]	I	28	77.5	85.71% F 14.29% M	54% DA	2 years	10,71%
Torrens et al. 2011 *JOT* [14]	IV	17	76.2	80% F 20% M	47.2% R 52.9% L	2 years	0%
Yüksel et al. 2011 *JOT* [15]	IV	18	67.7	61.1% F 38.9% M	33.3% R 66.7% L	39.1 months	0%
Boons et al. 2012 *Clinic. Ort* [16].	I	25	79.9	92% F 8% M		12 months	8%

*JSES* Journal of Shoulder and Elbow Surgery, *JOT* Journal Orthopedic Trauma

To classify the fractures, all studies used the well-known system proposed by Neer in 1970 [17], which has good interobserver reproducibility (*k* = 0.66) and moderate interobserver reliability (*k* = 0.50) [18]. According to the Neer classification, there were 55 three-part fractures and 78 four-part fractures. With regard to laterality and dominant limb, studies did not report sufficient data to calculate mean values or percentages. Mean loss to follow-up in the articles analyzed was 5.81%, ascending to 10% in the studies by Olerud et al. [12, 13].

With respect to the indications for conservative treatment, all studies applied conservative treatment to three- and four-part fractures, defined as having displacement > 1 cm or angulation > 45°, according to the Neer criteria [17]. Van den Broek et al. [11], Olerud et al. [12], Torrens et al. [14], and Yüksel et al. [15] treated in this way all three-part PHFs, while Van den Broek et al. [11], Olerud et al. [13], Torrens et al. [14], Yüksel et al. [15], and Boons et al. [16] used it in four-part PHFs.

All of the conservative treatment protocols included a period of immobilization with a sling or Velpeau for 2 to 4 weeks [11–16]. Subsequent rehabilitation was variable, with most studies using passive exercises at 2 weeks and active exercises at 4 (Table 3).

**Table 3:** Fracture type and treatment in included studies

Author	Fracture type	Treatment
Van den Broek et al. 2007 [11] *Arch Orthop Trauma Surg*	3- and 4-part Neer	Sling 4 weeks + passive ROM after 1 week
Olerud et al. 2011 *JSES* [12]	3-part Neer	Sling 2 weeks + rhb
Olerud et al. 2011 *JSES* [13]	4-part Neer	Sling 2 weeks + rhb
Torrens et al. 2011 *JOT* [14]	3 and 4-part Neer	Sling 3 weeks + rhb
Yüksel et al. 2011 *JOT* [15]	3 and 4-part Neer	Velpau 2 weeks + passive ROM after 2 weeks
Boons et al. 2012 *Clinic. Ort* [16].	4-part Neer	Shoulder in mobilizer 2 weeks + rhb

With regard to outcomes, all studies used the Constant score system (CS) [19, 20], which showed the following values: patients who suffered three-part fractures obtained an average CS of 64.5, pain of 11.1, range of movement (ROM) of 26.2, activities of daily living (ADL) of 15, and power of 13.9; and patients with four-part fractures obtained values of 54.9, 9.7, 21.7, 12.5, and 12.1, respectively.

In respect to subjective outcomes, there was greater variability in the type of assessment scales used. The most frequent was the visual analog scale (VAS) [21] and the Disabilities of the Arm, Shoulder and Hand Scoring System (DASH) [22]. Other studies used the EQ-5D Score [23], the Short Form Health Survey (SF-36) [24], and the Simple Shoulder Test (SST) [25] (Table 4).

**Table 4:** Objective and subjective outcomes achieved in included studies

Author	CS	Other scales	Subjective results
Van den Broek et al. 2007 [11] *Arch Orthop Trauma Surg*	3-part 82.1 4-part 80.6	Pain 11.3; ROM 30.3; Power 24.4; ADL 16.3 Pain 11.9; ROM 28; Power 23.8; ADL 17	VAS 1.7
Olerud et al. 2011 *JSES* [12]	3-part 58.4	Pain 11.2; ROM 20.1; Power 7.9; ADL 14.6	DASH 35 Eq-5d
Olerud et al. 2011 *JSES* [13]	4-part 49.6	Pain 11; ROM 20.1; Power 4.7; ADL 13.8	DASH 36.9 Eq-5d
Torrens et al. 2011 *JOT* [14]	3-part 54.64 4-part 33.66	Pain 9.44; Power 6.82; ADL 16.11; Pain 7; Power 2.66; ADL 8	VAS tarif 3-p 0.55 VAS tarif 4-p 0.3 Eq-5d
Yüksel et al. 2011 *JOT* [15]	3-part 63 4-part 50.6	Pain 12.5; ROM 23.5; Power 13.8; ADL 13; Pain 8.5; ROM 20.6; Power 10.2; ADL 11.5	SF-36
Boons et al. 2012 *Clinic. Ort* [16].	4-part 60	Pain 10; ROM 18; Power 19; ADL 12	VAS pain 25 VAS disability 31 SST

Concerning complications, 7.5% of patients experienced pseudoarthrosis, 21% malunion, 9% avascular necrosis, 4.5% post-traumatic arthrosis, 0% glenohumeral instability, and 0% torn rotator cuff (Table 5).

**Table 5:** Complications and rate of consolidation reported in included studies

Author	Complications	Consolidation
Van den Broek et al. 2007 [11] *Arch Orthop Trauma Surg*	1 patient with nonunion 10 patient with malunion at healing	92%
Olerud et al. 2011 *JSES* [12]	1 patient with nonunion 1 patient with impingement resulting from a malunited greater tubercle 2 patients with signs of minor AVN 1 patient post-traumatic osteoarthritis	96%
Olerud et al. 2011 *JSES* [13]	1 patient nonunion 1 patient without bony contact after one month was operated 3 patients with signs of AVN 5 patients post-traumatic osteoarthritis	96%
Torrens et al. 2011 *JOT* [14]	1 patient with stiffness 17 patient with malunion at healing	100%
Yüksel et al. 2011 *JOT* [15]	3 patients with nonunion 5 patients with signs of AVN	84%
Boons et al. 2012 *Clinic. Ort* [16].	2 patients had AVN of the head 3 patients had a nonunion of the four-part fracture 1 patient was operated on 13 months to hemiarthroplasty	80%

*AVN* avascular necrosis

Only one study was rated with high risk of bias because less than 6 out of the 12 criteria were met, as suggested by Furlan et al. [10].

## Discussion

Treating patients with a PHFs presents several challenges to orthopedic surgeons since imaging analysis with simple radiographs can be complex as demonstrated by the low kappa index of interobserver reliability and intraobserver reproducibility [17, 26–29]. there is no consensus on treatment indications [30], and it can be difficult to obtain and maintain an adequate reduction of the fracture in bones that are frequently osteoporotic [31].

In practice, it is customary to use nonoperative treatment for minimally displaced fractures according to the Neer criteria (< 1 cm displacement or < 45° angulation) [17]. One of our review questions consisted of determining which type of displaced fractures, frequently considered for surgical treatment, would benefit from a more conservative approach, which is inherently associated with fewer complications and risks [32]. In our analysis of the papers included in this review, we observed a significative difference between the outcomes for the three-part fractures compared with the four-part fractures.

Good results of orthopedic treatment in minimal displaced or two-part FHPs are widely known to the community [8, 14, 33–35]. The controversy around conservative versus surgical treatment has generally been raised with the indications for three- and four-part fractures, where heterogeneous results have contributed to the lack of consensus among orthopedic surgeons. Advances in osteosynthesis and shoulder prosthesis have made surgery a more appealing option, leading to its increased use for these types of fractures. Three-part PHFs made up 41% of the fractures studied, and acceptable results were obtained with a rate of consolidation of 96%, avascular necrosis (AVN) of 7%, malunion of 27%, and post-traumatic osteoarthritis of 1.8%. The mean Constant score in the studies reporting values for these fracture subgroups [11, 12, 14, 15] was 64.5. On the other hand, four-part PHFs made up 59% of the fractures studied. These fractures achieved worse results with a rate of consolidation of 90%, AVN of 10%, malunion of 17%, and post-traumatic osteoarthritis of 6%. We observed a mean Constant score of 54.9. All this supports a more aggressive treatment in this type of fractures in order to improve functional outcomes. It is worth mentioning that in Olerud’s studies [12, 13], the patients who were unable to achieve the test position of 90° were assigned a strength score of 0, following the recommendations of Constant [20]. This strict approach may have ended in an underestimation of Olerud’s results for the Constant score compared with previous studies.

The reason for the discrepancy between the Constant score versus the Eq-5D, DASH, and pain score is unclear. A possible explanation may be that the Constant score is not self-reported and is therefore less sensitive to subjectively experienced, yet important, differences in outcomes [36].

With regard to the range of motion and Constant score, we found differences between three- versus four-part fractures. The former [11, 12, 14, 15] showed mean pain of 11.1, ROM of 26.2, ADL of 15, power of 13.9, and Constant score of 64.5, while the latter showed values of 9.7, 21.7, 12.5, 12.1, and CS of 54.9, respectively [11, 13–16].

Although the outcomes achieved with conservative treatment for three- and four-part fractures were worse than those obtained for minimally displaced and two-part fractures [37], the surgical alternative is not risk-free. The rate of complications is high [38], and in surgeries using locking plates, it can rise to 70% [5]. The increased risk of complications might be acceptable if balanced against a guarantee of better functional outcomes than with conservative treatment, but this is not always possible in three- and four-part fractures [39].

In terms of the imaging studies of the PHFs, 50% of the studies used only simple radiographic projections, while the rest employed computerized tomography (CT) imaging to complete the diagnosis. Currently, classifying complex PHFs without CT, which is generally available in all hospital emergency departments, implies some risk of selection bias, as evaluation of radiographic images alone is imprecise [18, 26–29].

About conservative treatment protocols, and even rehabilitation, the scarce interest among orthopedic surgeons for this treatment modality is well-known once it is indicated. All studies employed a shoulder immobilizer (sling, Velpau) during 2 to 6 weeks followed by a rehabilitation program that began with passive exercises at 1 or 2 weeks after immobilization. Better findings at the evaluation of the Constant score happened in treatment protocols that began before 2 weeks of passive exercises as we have observed at Van den Broek et al. [11] who obtained CS for three-part PHFs of 82.1 and four-part PHF of 80.6.

This systematic review is the first after the PROFHER study [8], which generated considerable controversy by concluding that there was no difference in clinical outcomes between surgical versus conservative treatments of displaced PHFs. We did not include this study in our review because it did not fulfill the inclusion criteria. This study had some questionable characteristics, such as the selection of patients based on the criteria of the participating orthopedic surgeons, who were allowed to exclude patients whom they considered had a clear indication for surgery. Moreover, treatment protocols varied by center, complicating comparison. Nevertheless, the PROFHER study has undoubtedly marked a point of inflexion due to the evidence it provided on the non-superiority of surgical treatment of PHFs.

To our knowledge, the present systematic review is the second on conservative treatment of PHFs, updating the review published by Iyengar et al. in 2011 [33] and including 5 more recent studies. To increase the eligible sample size, we included all studies providing data on cohorts of PHFs that received conservative treatment and met our inclusion criteria, regardless of whether or not they were compared with surgical treatment. It was important to highlight the low number of studies included in our review since other studies did not analyze by subgroups and reported raw data of all displaced PHFs.

The pooled rate of consolidation following conservative treatment in the included studies was 92.5%. This high percentage shows that PHFs tend to consolidate, even in patients with poor bone quality [33]. It would be difficult to improve these results surgically, although where surgery could prove itself superior would be in the rate of malunion. Although criteria for diagnosing this complication were not always specified, and included studies reported very heterogeneous percentages, the rate of malunion in the series of Okike et al. [35] stood at 40% following surgery. The counterpoint is the relatively high risk of complications with surgical treatment. Rangan et al. [8] reported that 28.8% of patients treated surgically experienced complications, compared with 18.4% of those treated conservatively.

One frequent complication of conservative treatment of PHFs is loss of mobility. While this limitation could be used to argue in favor of surgical treatment, authors such as Olerud et al. [12] compared surgery with locking plates versus non-operative treatment in three-part fractures, achieving ranges of flexion of 120°, abduction of 114°, and a Constant score of 61 in the surgery group, compared with respective values of 111°, 106°, and 58.4 in the non-operative group—differences that were neither statistically significant nor clinically relevant.

With regard to avascular necrosis, our review found a mean rate of 9%. The correlation between risk of necrosis and severity of the fracture is well-known [40, 41], so the low rate reported in included studies is noteworthy, especially considering that the fractures included were three- or four-part fractures. Perhaps one of the reasons is that some studies only reported clinically symptomatic necrosis. Another reason could be that the mean follow-up of 32 months was not long enough to detect some cases with a later presentation. Studies have documented the manifestation of necrosis up to 3.5 years after the fracture [42].

This review is limited by the weaknesses of its included studies. Our work includes only six studies because the others did not include data by subgroups of fractures with regard to outcomes on range of motion, assessment scales, and complications, as the overall results may be deceptive. Moreover, the inclusion of retrospective studies always confers the risk of selection bias, as injuries treated nonoperatively tend to be less severe and therefore have better outcomes. Furthermore, it is difficult to compare results between studies using different assessment scales.

Focusing on the risk of bias, 1 of 6 studies had high risk of bias. It did not report about randomization, allocated interventions, and groups similar at baseline. The remaining 5 studies had a low risk of bias (Table 6).

**Table 6:**
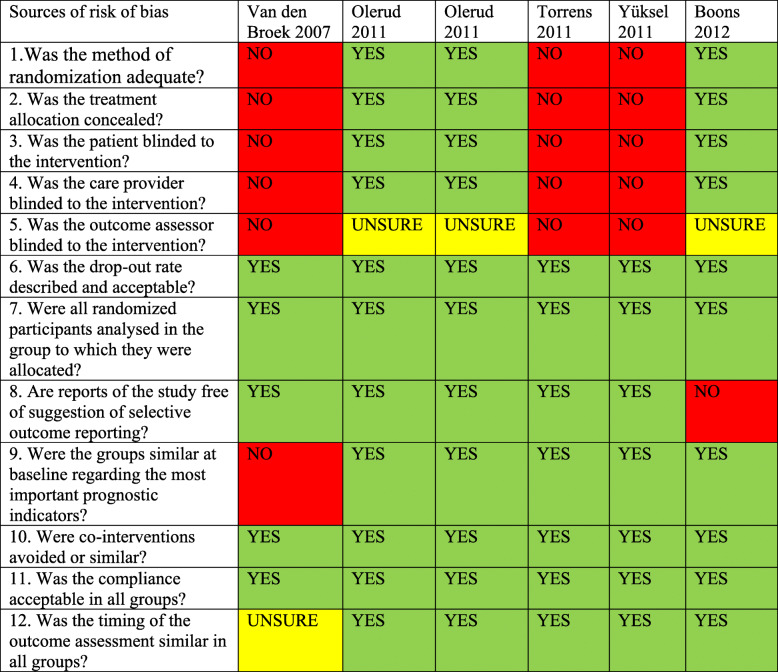
Sources of risk of bias according to Furlan et al. [10]

Future research is needed to improve knowledge around the possibilities of conservative treatment of PHFs, performing studies of subgroups of fractures and comparing diverse treatment protocols.

## Conclusion

Our study shows that most three-part PHFs can be treated conservatively, with a high rate of consolidation, fair–good functional outcomes in accordance with the severity of the fracture and few complications compared to surgical treatment. Conservative treatment for this type of fracture could be preferable in elderly patients with PHFs, as they should obtain acceptable functional outcomes and good control of pain while avoiding the potential risks of surgery. If patient functional expectations are high and have few comorbidities, more aggressive treatment could be indicated.

Otherwise, orthopedic treatment of four-part PHFs provided a high rate of consolidation with few complications, but worse functional results despite a less rate of malunion than three-part PHFs. This modality of treatment is advisable to four-part PHFs with low functional expectation or with severe comorbidities because poorer results are expected.

About conservative treatment protocols, we observed that an early start of passive exercises achieved better results and should be recommended.

## Data Availability

Data sharing is not applicable to this article as no datasets were generated or analyzed during the current study.

## Abbreviations

PHF: Proximal humeral fracture
MeSH: Medical subject headings
CS: Constant score
ROM: Range of movement
ADL: Activities of daily living
VAS: Visual analog scale
DASH: Disabilities of the Arm, Shoulder, and Hand Scoring System
SF-36: Short Form Health Survey
SST: Simple Shoulder Test
AVN: Avascular necrosis
CT: Computerized tomography

## References

[1] Bengner U, Johnell O, Redlund-Johnell I (1988). Changes in the incidence of fracture of the upper end of the humerus during a 30-year period. A study of 2125 fractures. Clin Orthop Relat Res.

[2] Court-Brown CM, Caesar B (2006). Epidemiology of adult fractures: a review. Injury.

[3] Zyto K (1998). Non-operative treatment of comminuted fractures of the proximal humerus in elderly patients. Injury..

[4] Edelson G, Safuri H, Salami J (2008). Natural history of complex fractures of the proximal humerus using a three-dimensional classification system. J Shoulder Elb Surg.

[5] Sudkamp N, Bayer J, Hepp P (2009). Open reduction and internal fixation of proximal humeral fractures with use of the locking proximal humerus plate. Results of a prospective, multicenter, observational study. J Bone Joint Surg Am.

[6] Williams GR, Wong KL (2000). Two-part and three-part fractures: open reduction and internal fixation versus closed reduction and percutaneous pinning. Orthop Clin North Am.

[7] Clavert P, Adam P, Bevort A (2010). Pitfalls and complications with locking plate for proximal humerus fracture. J Shoulder Elb Surg.

[8] Rangan A, Handoll H, Brealey S, PROFHER Trial Collaborators (2015). Surgical vs nonsurgical treatment of adults with displaced fractures of the proximal humerus: the PROFHER randomized clinical trial. JAMA..

[9] Moher D, Liberati A, Tetzlaff J, Altman DG (2009). The PRISMA group. Preferred reporting items for systematic reviews and meta-analyses: the PRISMA statement. PLoS Med.

[10] Furlan D et al.2009 updated method guidelines for systematic reviews in the Cochrane Back Review Group. Spine. 2009;34(18):1929–41. 10.1097/BRS.0b013e3181b1c99f. 10.1097/BRS.0b013e3181b1c99f 19680101

[11] Van den Broek CM, van den Besselaar M, Coenen JM, Vegt PA (2007). Displaced proximal humeral fractures: intramedullary nailing versus conservative treatment. Arch Orthop Trauma Surg.

[12] Olerud P, Ahrengart L, Ponzer S (2011). Internal fixation versus nonoperative treatment of displaced 3-part proximal humeral fractures in elderly patients: a randomized controlled trial. J Shoulder Elb Surg.

[13] Olerud P, Ahrengart L, Ponzer S (2011). Hemiarthroplasty versus nonoperative treatment of displaced 4-part proximal humeral fractures in elderly patients: a randomized controlled trial. J Shoulder Elb Surg.

[14] Torrens C, Corrales M, Vilà G (2011). Functional and quality-of-life results of displaced and nondisplaced proximal humeral fractures treated conservatively. J Orthop Trauma.

[15] Yüksel HY, Yılmaz S, Akşahin E, Celebi L, Muratli HH, Biçimoğlu A (2011). The results of nonoperative treatment for three- and four-part fractures of the proximal humerus in low-demand patients. J Orthop Trauma.

[16] Boons HW, Goosen JH, van Grinsven S, van Susante JL, van Loon CJ (2012). Hemiarthroplasty for humeral four-part fractures for patients 65 years and older: a randomized controlled trial. Clin Orthop Relat Res.

[17] Neer CS (1970). Displaced proximal humeral fractures. I. Classification and evaluation. J Bone Joint Surg Am.

[18] Sidor ML, Zuckerman JD, Lyon T (1993). The Neer classification system for proximal humeral fractures. An assessment of interobserver reliability and intraobserver reproducibility. J Bone Joint Surg Am.

[19] Constant CR, Gerber C, Emery RJ (2008). A review of the Constant score: modifications and guidelines for its use. J Shoulder Elb Surg.

[20] Constant CR, Murley AH (1987). A clinical method of functional assessment of the shoulder. Clin Orthop Relat Res.

[21] Price DD, Mc Grath PA (1983). The validation of visual analogue scales as ratio scale measures for chronic and experimental pain. Pain.

[22] Hudak PL, Amadio PC, Bombardier C (1996). Development of an upper extremity outcome measure: the DASH (disabilities of the arm, shoulder and hand) [corrected]. The upper extremity collaborative group (UECG). Am J Ind Med.

[23] Dolan P, Gudex C, Kind P (1996). The time trade-off method: results from a general population study. Health Econ.

[24] Ware JE, Sherbourne CD (1992). The MOS 36-item short-form health survey (SF-36): I. conceptual framework and item selection. Med Care.

[25] Godfrey J, Hamman R, Lowenstein S (2007). Reliability, validity, and responsiveness of the simple shoulder test: psychometric properties by age and injury type. J Shoulder Elb Surg.

[26] Bernstein J, Adler LM, Blank JE (1996). Evaluation of the Neer system of classification of proximal humeral fractures with computerized tomographic scans and plain radiographs. J Bone Joint Surg Am.

[27] Kristiansen B, Andersen UL, Olsen CA (1988). The Neer classification of fractures of the proximal humerus. An assessment of interobserver variation. Skelet Radiol.

[28] Sallay PI, Pedowitz RA, Mallon WJ (1997). Reliability and reproducibility of radiographic interpretation of proximal humeral fracture pathoanatomy. J Shoulder Elb Surg.

[29] Siebenrock KA, Gerber C (1993). The reproducibility of classification of fractures of the proximal end of the humerus. J Bone Joint Surg Am.

[30] Handoll HHG, Ollivere BJ. Interventions for treating proximal humeral fractures in adults. Cochrane Database Syst Rev. 2010;12:1 (Art. No. CD000434). 10.1002/14651858.CD000434.pub2. 10.1002/14651858.CD000434.pub2 21154345

[31] Lanting B, MacDermid J, Drosdowech D (2008). Proximal humeral fractures: a systematic review of treatment modalities. J Shoulder Elb Surg.

[32] Roberson TA, Granade CM, Hunt Q (2017). Nonoperative management versus reverse shoulder arthroplasty for treatment of 3- and 4-part proximal humeral fractures in older adults. J Shoulder Elb Surg.

[33] Iyengar JJ, Devcic Z, Sproul RC (2011). Nonoperative treatment of proximal humerus fractures: a systematic review. J Orthop Trauma.

[34] Court-Brown CM, Cattermole H, McQueen MM (2002). Impacted valgus fractures (B1.1) of the proximal humerus. The results of non-operative treatment. J Bone Joint Surg (Br).

[35] Okike K, Lee OC, Makanji H, Morgan JH, Harris MB, Vrahas MS (2015). Comparison of locked plate fixation and nonoperative management for displaced proximal humerus fractures in elderly patients. Am J Orthop (Belle Mead NJ).

[36] Harvie P, Carr AJ (2004). Outcome measures in orthopedics and orthopedic trauma.

[37] Lill (2001). Conservative treatment of dislocated proximal humeral fractures. Zentralbl Chir.

[38] Owsley KC, Gorczyca JT (2008). Fracture displacement and screw cutout after open reduction and locked plate fixation of proximal humeral fractures. J Bone Joint Surg Am.

[39] Kontakis G, Koutras C, Tosounidis T (2008). Early management of proximal humeral fractures with hemiarthroplasty: a systematic review. J Bone Joint Surg (Br).

[40] Bogner R, Hübner C, Matis N (2008). Minimally-invasive treatment of three- and four-part fractures of the proximal humerus in elderly patients. J Bone Joint Surg (Br).

[41] Kralinger F, Unger S, Wambacher M (2009). The medial periosteal hinge, a key structure in fractures of the proximal humerus: a biomechanical cadaver study of its mechanical properties. J Bone Joint Surg (Br).

[42] Lee CK, Hansen HR (1981). Post-traumatic avascular necrosis of the humeral head in displaced proximal humeral fractures. J Trauma.

